# Chemical Stimulation of Rodent and Human Cortical Synaptosomes: Implications in Neurodegeneration

**DOI:** 10.3390/cells10051174

**Published:** 2021-05-12

**Authors:** Faraz Ahmad, Yu Jing, Albert Lladó, Ping Liu

**Affiliations:** 1 Department of Anatomy, University of Otago, Dunedin 9016, New Zealand; faraz.ahmad@otago.ac.nz (F.A.); rena.jing@otago.ac.nz (Y.J.); 2 Alzheimer’s Disease and Other Cognitive Disorders Unit, Neurology Department, Hospital Clínic, Institut Investigació Biomèdica August Pi i Sunyer (IDIBAPS), 08036 Barcelona, Spain; allado@clinic.cat

**Keywords:** glycine, KCl, NMDAR, depolarization, rolipram, forskolin, tetraethyl ammonium, PS19 mice, latrunculin A, radio-labelled

## Abstract

Synaptic plasticity events, including long-term potentiation (LTP), are often regarded as correlates of brain functions of memory and cognition. One of the central players in these plasticity-related phenomena is the α-amino-3-hydroxy-5-methylisoxazole-4-propionate receptor (AMPAR). Increased levels of AMPARs on postsynaptic membranes thus constitute a biochemical measure of LTP. Isolated synaptic terminals (synaptosomes) are an excellent ex vivo tool to monitor synaptic physiology in healthy and diseased brains, particularly in human research. We herein describe three protocols for chemically-induced LTP (cLTP) in synaptosomes from both rodent and human brain tissues. Two of these chemical stimulation protocols are described for the first time in synaptosomes. A pharmacological block of synaptosomal actin dynamics confirmed the efficiency of the cLTP protocols. Furthermore, the study prototypically evaluated the deficiency of cLTP in cortical synaptosomes obtained from human cases of early-onset Alzheimer’s disease (EOAD) and frontotemporal lobar degeneration (FLTD), as well as an animal model that mimics FLTD.

## 1. Introduction

Synapses, the communication points between neurons, elicit tremendous plasticity and undergo dynamic short- and long-term modifications. Activity-dependent alterations in synaptic plasticity, such as long-term potentiation (LTP) and long-term depression (LTD), are thought to represent the cellular basis of learning and memory [1,2,3]. Among the most well-characterized molecular targets at the center of synaptic plasticity-related phenomena are the α-amino-3-hydroxy-5-methylisoxazole-4-propionate (AMPA) types of glutamatergic receptors. AMPA receptors (AMPARs) are rapidly cycled between intracellular pools and synaptic surfaces during LTP and LTD [4,5,6,7]. In fact, both electrical and chemical (chemical LTP or cLTP) means of LTP induction have been shown to result in rapid trafficking of AMPARs at the plasmalemmal surfaces of the synaptic terminals [8,9,10].

Synaptosomes are biochemically isolated subcellular preparations of presynaptic terminals in close apposition to postsynaptic densities [11,12,13]. These preparations can be conveniently obtained from post-mortem brain tissues, and are excellent ex vivo models to recapitulate multiple aspects of basal and activity-induced synaptic phenomena. In fact, because of the preservation of most enzymatic and metabolic activities, synaptosomes serve as efficient “halfway house” tools between neurochemistry and electrophysiology [11,12,13,14,15]. It is worth noting that synaptosomes are being established as a prominent research tool to understand the mechanisms of synaptic dysfunction in diseased human brains, wherein the acquisition of acute slices and cultures are technically challenging [11,13,15]. Importantly, viable synaptosomes can be obtained from post-mortem human brain tissues with significant post-mortem delays and storage times [16,17,18,19]. In recent years, synaptosomes from AD brains have been utilized to study different aspects of synaptic physiology, such as neurotransmitter release and uptake [20,21], disease pathogenesis [22,23,24,25], protein translation [26,27], actin dynamics [28,29], protein and lipid signaling cascades [26,30,31,32,33] and bioenergetic functions [34,35,36].

The present study aimed to further establish the utility of synaptosomal preparations from human and rodent brain tissues in studying cLTP events using three different chemical stimulation protocols with AMPAR insertion at postsynaptic sites as a common readout. The first chemical stimulation protocol involved depolarization mediated by high extracellular K^+^ following the priming of N-methyl-D-aspartate receptors (NMDARs) with glycine. This cLTP protocol has been employed in ex vivo synaptosomes coupled with various methods for the assessment of surface-expressed AMPARs, including liquid scintillation [37], surface biotinylation followed by immunoblotting [38] and immunolabelling followed by fluorescence-based sorting [39,40,41]. The second stimulation protocol is based upon the coincubation of rolipram and forskolin, a phosphodiesterase 4 (PDE4) inhibitor and an adenylate cyclase activator respectively, to assess NMDAR-dependent LTP [42,43,44,45,46,47]. The third protocol utilizes a potassium channel blocker tetraethyl ammonium (TEA), which induces membrane depolarization and a consequent burst of calcium spikes resulting in an NMDAR-independent form of LTP [48,49,50,51,52]. Although the latter two protocols have been employed in cell cultures and acute slices, for the first time, the present study applied both methods to synaptosomes obtained from either rodent and human brain tissues for the induction of cLTP. It should be noted, however, that there are reports of employing rolipram and/or forskolin for the stimulation of presynaptic neurotransmitter release in isolated synaptosomes [53,54,55,56].

The second aim of this study was to obtain a proof-of-principle of the cLTP protocols in ex vivo synaptosomes, by evaluating the efficacy of the three protocols via a pharmacological blockade of synaptosomal actin dynamics. Actin is a cytoskeletal protein forming the integral framework maintaining both structural and functional aspects of synaptic physiology [57,58,59]. Actin dynamics, the interconversion between its monomeric globular form (G-actin) and its polymerized filamentous form (F-actin), plays a fundamental role in controlling the size, number and morphology of neuronal dendritic spines. It has been well documented that both pre- and post-synaptic functions are critically dependent on actin dynamics [60,61,62,63,64]. We therefore determined the effects of blocking actin dynamics on cLTP-induced changes in surface-exposed AMPARs in synaptosomes using latrunculin A [65,66,67,68].

Synapse loss and synaptic dysfunction closely correlate with cognitive deficits in pathophysiological conditions across the whole developmental spectrum of the central nervous system (CNS), ranging from early life insults [69,70,71] to neurodegenerative disorders such as Alzheimer’s disease (AD) and frontotemporal lobar degeneration (FTLD) [72,73,74,75]. Moreover, there is recent evidence indicating the dysfunction of synaptic cytoskeletal actin dynamics in neurodegeneration [28,29,76,77,78,79,80]. The third aim of the study was therefore to obtain the proof-of-concept of the utility of synaptosomes in the determination of cLTP changes in neurodegenerative disorders using post-mortem brain tissues from human subjects of early-onset AD (EOAD) and frontotemporal lobar degeneration (FTLD), and PS19 mice bearing human microtubule-associated protein tau P301S mutation, which mimics human FTLD [81,82].

## 2. Materials and Methods

### 2.1. Human Tissues

Human brain tissues were obtained from the Neurological Tissue Bank of Hospital Clínic-IDIBAPS BioBank in Barcelona, Spain. All tissue collection protocols were approved by the Ethics Committee of Hospital Clínic, Barcelona (October 2018) and informed consent was obtained from all families. The unfixed snap-frozen superior frontal gyrus was obtained from EOAD, FTLD and neurologically normal (NOR) cases (*n* = 5/group). The neurologically normal cases were defined by the absence of any history of neurological and psychiatric diseases and the absence of any neuropathological findings from a detailed analysis of the brain by an independent neuropathologist. All diseased cases fulfilled the neuropathological consensus criteria for AD or FTLD. EOAD was defined as AD with age at onset before 65 years. There were no significant differences between groups in terms of the age and post-mortem delay (Table 1). The experimenters were blind to the grouping information.

### 2.2. Animals and Tissue Collection

Male P301S tau transgenic (PS19) mice (B6;C3-Tg(Prnp-MAPT*P301S)PS19Vle/J; stock number: 008169) and C57BL/6J female mice were crossed to produce the offspring of PS19 mice and wildtype (WT) littermates that were confirmed by tail tip genotyping. The present study used 8 months old male PS19 and WT mice, as well as 5 months old male Sprague-Dawley rats. All animals were maintained on a 12-h light/dark cycle with *ad libitum* access to food chow and water. All animals were anesthetized with sodium pentobarbitone and transcardially perfused with ice-cold saline. The brain from each animal was rapidly removed and transferred to saline on ice. Brain tissues (cortices) were then freshly dissected on ice from each hemisphere and immediately snap-frozen and stored at −80 °C [83,84]. All experimental procedures were carried out in accordance with the regulations of the University of Otago Committee on Ethics in the Care and Use of Laboratory Animals and New Zealand legislature (Ethics Protocol No. AUP-95-18 and AUP-80-17).

### 2.3. Drugs, Reagents and Antibodies

Phosphatase inhibitor cocktail IV (ab201115) and EDTA-free protease inhibitor cocktail (4693159001) were from Abcam (Cambridge, MA, USA) and Roche Diagnostics (Mannheim, Germany), respectively. [^3^H]-AMPA (DL-α-[5-methy-3H)) was obtained from Perkin Elmer (Boston, MA, USA; NET833250UC; 58.1 Ci/mmol). Potassium thiocyanate (KSCN; 207799), tetraethyl ammonium (TEA; T-2265) and latrunculin A (L5163) were procured from Sigma-Aldrich (St. Louis, MO, USA). Forskolin (HB1348) was from HelloBio. Rolipram (ab120029) and primary antibodies against PSD-95 (rabbit; ab18258) and GAPDH (mouse; ab8245) were from Abcam (Cambridge, MA, USA). Fluorescent secondary antibodies (IRDye^®^ 800 CW donkey anti-rabbit IgG and IRDye^®^ 680 RD goat anti-mouse IgG) were from LI-COR Biosciences (Lincoln, NE, USA). Other regents used in the study were of analytical grade and purchased either from Thermo Fisher Scientific or Sigma-Aldrich.

### 2.4. Preparation of Synaptosomes

Synaptosomes from either human or rodent brain tissues were prepared as described in our previous studies [26,30,85]. Briefly, tissues were homogenized using a Potter-*Elvehjem* tissue grinder (Duran Wheaton Kimble, Mexico; 358034) in 10 volumes of ice-cold homogenization buffer containing 5 mM HEPES (pH 7.4) and 0.32 M sucrose supplemented with protease and phosphatase inhibitor cocktails. The homogenate was centrifuged at 1500× *g* for 10 min at 4 °C to obtain a crude nuclear pellet (containing nuclei and cellular debris), which was discarded. The supernatant obtained was further centrifuged at 12,000× *g* for 15 min at 4 °C to obtain a crude synaptosomal pellet containing mitochondria and myelin. This crude synaptosomal pellet was resuspended in 5 mM Tris buffer at pH 7.4 containing 0.32 M sucrose supplemented with protease and phosphatase inhibitor cocktails. The resuspended synaptosomal pellet was then subjected to fractionation on a discontinuous sucrose gradient (equal volumes of 0.85 M, 1.0 M and 1.2 M sucrose) at 85,000× *g* for 1.5 hr at 4 °C to separate out the contaminating mitochondria and myelin fractions from the synaptosomes. The purified synaptosomes were collected from the interface between the 1.0 and 1.2 M sucrose layers, and then subjected to two washes, first with 5 mM Tris buffer (pH 8.1) and second with homogenization buffer, at 12,000× *g* for 15 min at 4 °C. The washed synaptosomes were resuspended in homogenization buffer for further analyses. An aliquot of synaptosomes from each sample was used for the quantification of the protein content using Bradford assay. Of note, discontinuous gradient-based subcellular fractionation has been shown to generate intact and enriched synaptic terminals from both human and rodent brain tissues by us and others [19,26,30,85,86,87].

### 2.5. Immunoblotting

Immunoblotting was performed to assess the purity of the synaptosomes by evaluating the enrichment of PSD-95, a synaptic protein marker. For this, homogenates and synaptosomal samples were mixed with gel loading buffer (containing 50 mM Tris-HCl at pH 6.8, 10% SDS, 10% glycerol, 10% 2-mercaptoethanol and 2 mg/mL bromophenol blue) and were heated at 95 °C for 5 min. Ten μg protein per sample was loaded on a Criterion^TM^ XT 4–12% gradient SDS-PAGE gel (BioRad; 3450124), and electroblotted onto a nitrocellulose membrane as detailed in our publication [88]. Following blocking with 5% BSA in Tris-buffered saline at pH 7.4 containing 1% Tween 20 (TBST), the membrane was immunostained with primary antibodies against PSD-95 and housekeeping protein GAPDH. Fluorescently labelled secondary antibodies were used to generate immunoreactive signals. The signals were detected using the Odyssey Infrared System (LI-COR Biosciences; Lincoln, NE, USA) at 700 and 800 nm, and the analysis of immunoreactive signals was performed using ImageStudioLite (LI-COR Biosciences; Lincoln, NE, USA).

### 2.6. cLTP Protocol 1: Glycine Priming—KCl Depolarization (gK)

Synaptosomal NMDAR priming and stimulation were carried out based upon the methodologies from previous publications with modifications [37,38,39,40,41,89]. Briefly, synaptosomes (200 μg protein) were resuspended in duplicates with one set in buffer A1 (containing in mM; 120 NaCl, 3 KCl, 2 CaCl_2_, 2 MgCl_2_, 15 glucose, 15 HEPES; pH 7.4) and the other set in buffer B1 (containing in mM; 125 NaCl, 2 CaCl_2_, 5 KCl, 30 glucose, 10 HEPES at pH 7.4, 0.02 bicuculline and 0.001 strychnine), and pre-incubated at 37 °C for 10 min. A1 synaptosomes constituted the synaptosomes with basal activity, while B1 synaptosomes underwent cLTP. Glycine (500 μM) was added to B1 synaptosomes along with fresh bicuculline (0.02 mM) and strychnine (0.001 mM) to prime NMDARs for 15 min at 37 °C. An equal volume of buffer A1 was added to A1 synaptosomes for 15 min at 37 °C. Depolarization-induced stimulation of NMDARs was performed by a steep increase in KCl to a final [K^+^]_extracellular_ of 50 mM, via the addition of buffer C1 (containing in mM; 50 NaCl, 2 CaCl_2_, 100 KCl, 30 glucose, 0.5 glycine and 10 HEPES at pH 7.4, 0.02 bicuculine and 0.001 strychnine). Depolarization was proceeded for a duration of 60 min at 37 °C. A1 Synaptosomes (mock-stimulated) were supplemented with an equal volume of buffer A1 and incubated for the same duration of 60 min at 37 °C. cLTP in synaptosomes was measured as changes in the surface expression of AMPARs, evaluated by [^3^H]-AMPA binding (Section 2.10).

### 2.7. cLTP Protocol 2: Rolipram—Forskolin (RF)

This protocol takes advantage of the fact that a sustained increase of cyclic adenosine monophosphate (cAMP) level in the presence of forskolin (an adenylate cyclase activator) and rolipram (PDE4 inhibitor) results in the induction of cLTP, as shown earlier in acute slices [42,43,44,90], primary neurons [45,46,47,91,92] and organotypic slice cultures [93,94]. Here, we extended this cLTP protocol to ex vivo stimulation of synaptosomes with minor modifications. Briefly, synaptosomes (200 μg protein) were resuspended in duplicates, one set in buffer A2 (containing in mM; 120 NaCl, 3 KCl, 2 CaCl_2_, 2 MgCl_2_, 15 glucose and 15 HEPES at pH 7.4) and the other in buffer B2 (containing in mM; 125 NaCl, 5 CaCl_2_, 5 KCl, 30 glucose and 15 HEPES at pH 7.4, 0.02 bicuculline and 0.001 strychnine) and pre-incubated at 37 °C for 10 min. B2 synaptosomes (that underwent cLTP) were treated with 50 μM forskolin and 0.1 μM rolipram for 30 min at 37 °C. An equal volume of buffer A2 was added to control mock-stimulated A2 synaptosomes for the same duration of 30 min at 37 °C. cLTP was calculated as increases in the surface expression of AMPARs, measured by [^3^H]-AMPA binding on synaptosomal membranes (Section 2.10).

### 2.8. cLTP Protocol 3: Tetraethyl Ammonium (TEA)

The third chemical stimulation of synaptosomes was carried out in the presence of TEA, a non-specific voltage-gated potassium channel blocker that has been used to induce NMDAR-independent cLTP in acute slices [44,48,49,50,51,52], primary neurons [66,95,96] and organotypic slice cultures [97,98]. Briefly, synaptosomes (200 μg protein) was resuspended in duplicates with one set in buffer A3 and the other in buffer B3, and then pre-incubated at 37 °C for 10 min. cLTP was induced in B3 synaptosomes by addition of 50 mM TEA at 37 °C for 15 min, while control mock-stimulated A3 synaptosomes were supplemented with an equal volume of buffer A3 for the same duration of incubation. cLTP was quantitated as changes in the levels of surface-exposed AMPARs, which was evaluated by [^3^H]-AMPA binding (Section 2.10).

### 2.9. Pharmacological Block of Actin Polymerization

Pharmacological blockade of actin polymerization has often been employed to evaluate its role in mediating plastic changes at the synapses induced by neuronal activity. Latrunculin A, a pharmacological agent that depolymerizes actin, has been commonly used to abolish LTP [66,68,99,100,101]. Of note, low concentrations of latrunculin A prevent actin polymerization without leading to substantial depolymerization of existing actin filaments, hence preserving the basal synaptic organization [66,101,102]. Latrunculin A at a concentration of 200 nM was used in the present study to depolymerize actin filaments without affecting the basal surface-exposed AMPAR levels. Pre-incubation with latrunculin A was carried out both for synaptosomes undergoing cLTP (B1, B2 or B3) and control unstimulated synaptosomes (A1, A2 or A3) at 37 °C for 30 min. Induction of cLTP (in B1, B2 or B3 synaptosomes) or mock-cLTP stimulation (for A1, A2 or A3 synaptosomes) was also performed in the presence of latrunculin A. Effects of latrunculin A on cLTP was again assessed by evaluating the changes in synaptosomal [^3^H]-AMPA binding (Section 2.10).

### 2.10. [^3^H]-AMPA Labelling

Exogenous radiolabeled [^3^H]-AMPA has been conveniently employed to evaluate changes in the surface-expressed AMPARs using both autoradiography and liquid scintillation spectroscopy [37,103,104,105]. [^3^H]-AMPA labelling of synaptosomes in the presence of KSCN was carried out as described previously [37,106,107,108]. KSCN was used because this thiocyanate chaotropic anion increases the efficiency of AMPA binding [106]. First, ex vivo cLTP (or mock-cLTP) in synaptosomes was culminated by addition of 1 mL of ice-cold binding buffer (containing in mM; 100 Tris-acetate at pH 7.4, 0.1 EGTA and 100 KSCN). Following centrifugation at 12,000× *g* for 15 min at 4 °C, the synaptosomal pellets were resuspended and incubated in binding buffer on ice for 30 min. Synaptosomal AMPA-labeling was carried in binding buffer containing 25 nM [^3^H]-AMPA on ice for 60 min. Binding of radiolabeled AMPA was terminated by centrifugation at 18,000× *g* at 4 °C for 10 min, followed by a superficial wash of the pellet with ice-cold binding buffer. Synaptosomal pellets were then solubilized in 0.2 N NaOH and the bound radioactivity was counted by liquid scintillation spectrometry on a Perkin Elmer TriCarb 2910 TR liquid scintillator (Downers Grove, IL, USA). Non-specific binding of AMPA was determined in the presence of 100 mM glutamate [107,108]. To account for any variable retention of stimulated and unstimulated paired sets of synaptosomes during the washing steps, protein content of the AMPA-labelled synaptosomes were evaluated using Bradford assay.

### 2.11. Statistical Analyses

[^3^H]-AMPA binding is represented as femtomoles per mg protein. Data were presented as mean ± standard error of mean (SEM). Statistically significant differences in [^3^H]-AMPA binding between cLTP-stimulated synaptosomes and their respective mock-stimulated control samples were evaluated using two-tailed paired Student’s *t*-test with a *p* < 0.05 considered to be significant. All analyses and representation of the data were performed using GraphPad Prism 9 software (San Diego, CA, USA).

## 3. Results

### 3.1. Biochemical Preparations of Synaptosomes Are Enriched in PSD-95

**Figure 1:**
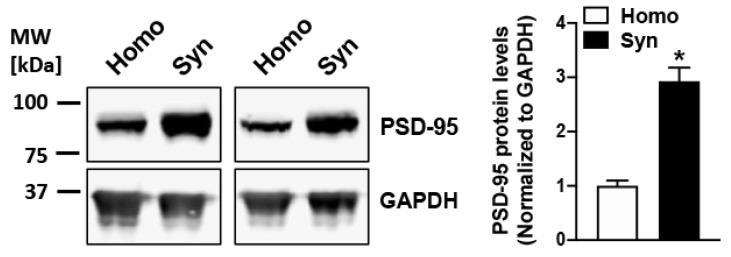
PSD-95 is enriched in rat cortical synaptosomes obtained by sucrose density gradient-based subcellular fractionation. Robust enrichment of synaptic marker protein PSD-95 is observed in synaptosomes (Syn) as compared to the respective starting homogenate (Homo) samples (*n* = 3), indicating a sturdy enhancement of isolated synaptic terminals in the synaptosomal fraction. * represents a statistical significance at *p* < 0.05.

We determined the protein levels of PSD-95 (a synaptic marker protein) in the rat cortical homogenates and synaptosomes isolated using a discontinuous sucrose gradient fractionation. In consistence with our previous studies [30,85], a robust 3-fold increase of PSD-95 was observed in synaptosomal samples when compared to the starting homogenates (*p* = 0.025; Figure 1), confirming appreciable enrichment of vesicular synaptic terminals in the our synaptosomal preparations.

### 3.2. Stimulation of Synaptosomes Results in AMPAR Mobilization at the Surface

**Figure 2:**
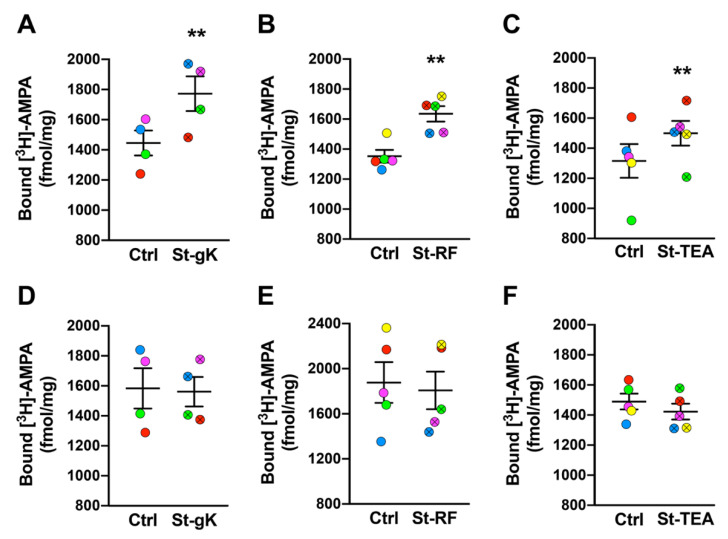
Chemically induced long-term potentiation (cLTP) in rat cortical synaptosomes (**A**–**C**) is abolished by latrunculin A (**D**–**F**). All three chemical stimulation protocols, glycine-KCl (St-gK; **A**), rolipram-forskolin (St-RF; **B**) and TEA (St-TEA; **C**), resulted in elevated surface AMPAR expression in rat cortical synaptosomes relative to their corresponding non-stimulation matched controls (Ctrl). However, latrunculin A (200 nM; a blocker of actin polymerization) completely abolished cLTP-induced insertion of AMPARs on rat synaptosomal membranes following the stimulations by glycine-KCl (**D**), rolipram-forskolin (**E**) and TEA (**F**). Results are expressed as mean (±SEM) bound [^3^H]-AMPA (*n* = 4–6 for each experiment). ** represents a statistical significance at *p* < 0.01. The respective pairs of stimulated and unstimulated synaptosomes are color coded in each panel.

Changes in plasmalemmal AMPARs induced by the three protocols of cLTP in ex vivo synaptosomes were evaluated using exogenous [^3^H]-AMPA binding. Synaptosomes from rat brain cortices primed with glycine and depolarized with high extracellular K^+^ elicited a significant increase (23%; *p* = 0.004) in surface-exposed AMPARs compared to the respective mock-stimulated (with no glycine and KCl treatments) controls (Figure 2A). Appreciable increases in surface-expressed AMPARs were also observed upon stimulations of synaptosomes with rolipram-forskolin (21%; *p* = 0.001 Figure 2B) when compared to the respective unstimulated synaptosomes (without treatment with rolipram-forskolin). Lastly, cLTP induction with TEA also resulted in increments in synaptosomal binding of [^3^H]-AMPA (15%; *p* = 0.005; Figure 2C) when compared to the respective mock-stimulated control synaptosomes (without treatment with TEA).

### 3.3. Latrunculin A Abolishes cLTP-Induced Stimulation in AMPAR Surface Expression

Actin dynamics is a primary event in mediating plasticity-induced responses at the synapses. Hence, we proceeded to determine the effects of blocking actin polymerization on the changes in surface-exposed AMPARs induced by cLTP via all three chemical protocols. Synaptosomes were pre-treated with latrunculin A (200 nM) and their stimulation (or mock-stimulation) was also performed in the presence of latrunculin A. Importantly, the sustained presence of latrunculin A (and consequent failure to stimulate new actin depolarization) prevented the increases in surface-exposed AMPARs following the cLTP induction by all the three protocols; glycine-KCl (Figure 2D), rolipram-forskolin (Figure 2E) and TEA (Figure 2F).

### 3.4. cLTP Can Be Induced in Human Cortical Synaptosomes

**Figure 3:**
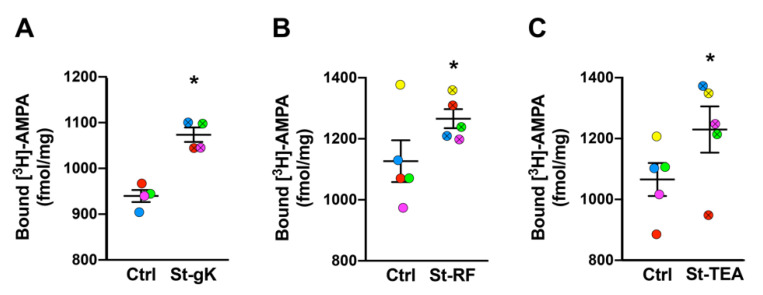
Long-term potentiation (LTP) is chemically induced in synaptosomes from superior frontal gyri of cognitively normal cases. All three chemical stimulation protocols, glycine-KCl (St-gK; **A**), rolipram-forskolin (St-RF; **B**) and TEA (St-TEA; **C**), resulted in increased levels of surface AMPARs relative to their corresponding non-stimulation matched controls (Ctrl). Results are expressed as mean (±SEM) bound [^3^H]-AMPA (*n* = 4–5 for each experiment). * represents a statistical significance at *p* < 0.05. The respective stimulated and unstimulated synaptosome pairs are color coded in each panel.

We next sought to extend the chemical stimulation protocols to synaptosomes from superior frontal gyri of cognitively normal human cases. Again, significant increases in surface AMPAR expression levels were induced by glycine-KCl (14%; Figure 3A), rolipram-forskolin (13%; Figure 3B) and TEA (15%; Figure 3C). Among the three cLTP protocols, the most consistent stimulation was achieved by glycine priming and KCl depolarization.

### 3.5. cLTP Is Impaired in Frontocortical Synaptosomes of EOAD and FTD Cases

**Figure 4:**
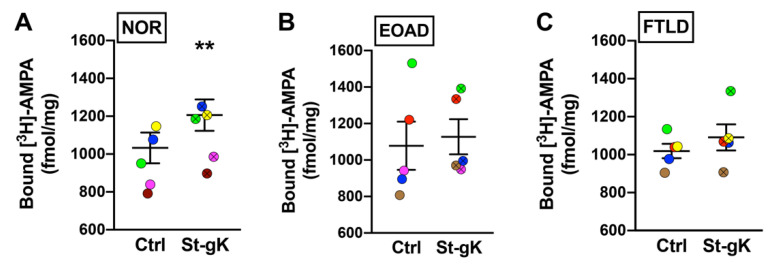
Chemically induced long-term potentiation (cLTP) is deficient in human cortical synaptosomes from patients with neurodegeneration. Surface AMPAR levels in cortical synaptosomes obtained from the normal (NOR), early-onset Alzheimer’s disease (EOAD) and frontotemporal lobar degeneration (FTLD) cases were assessed following glycine priming and KCl stimulation (St-gK). A robust cLTP was induced by St-gK relative to non-stimulation control (Ctrl) in synaptosomes from the normal cases (**A**), but not the EOAD (**B**) or FTLD (**C**) cases. Results are expressed as mean (±SEM) bound [^3^H]-AMPA (*n* = 5 for each experiment). ** represents a statistical significance at *p* < 0.01. The respective stimulated and unstimulated synaptosome pairs are color coded in each panel.

Using the glycine priming and KCl depolarization protocol, the present study determined the cLTP changes in synaptosomes from tissue samples of superior frontal gyri obtained from EOAD and FTLD cases relative to cognitively normal (NOR) subjects. Upon the evaluation of [^3^H]-AMPA labelling following priming with glycine and subsequent stimulation with KCl, a significant increase of surface AMPAR expression was observed in the synaptosomes from the normal cases (17%, *p* = 0.009; Figure 4A). However, the same protocol elicited a small but insignificant increase in surface AMPAR levels of the synaptosomes obtained from the EOAD (6%, *p* = 0.41; Figure 4B) or FTLD (7%, *p* = 0.11; Figure 4C) cases, indicating synaptic cLTP deficits in patients with neurodegenerative disorders. Of note, the basal (unstimulated) levels of surface-exposed AMPARs were not found to be significantly altered between the three groups.

### 3.6. cLTP Is Impaired in Cortical Synaptosomes of PS19 Mice

**Figure 5:**
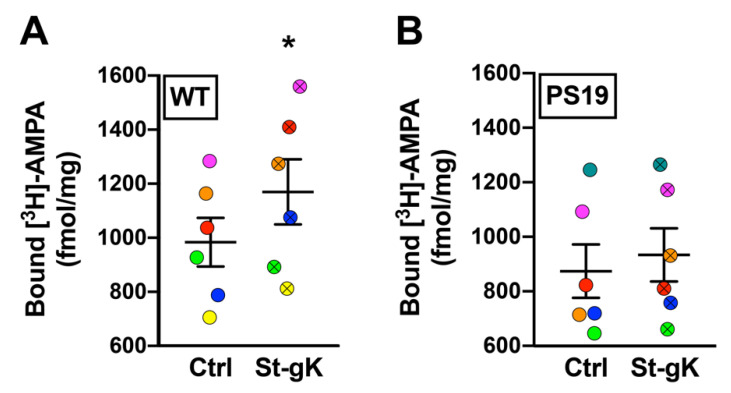
Chemically induced long-term potentiation (cLTP) is abolished in cortical synaptosomes of 8 months old PS19 mice. Surface AMPAR levels in cortical synaptosomes obtained from wildtype (WT) and PS19 mice were assessed following glycine priming and KCl stimulation (St-gK). A robust cLTP was induced in synaptosomes from WT mice (**A**), but not in PS19 mice (**B**). Results are expressed as mean (±SEM) bound [^3^H]-AMPA (*n* = 6 for each experiment). * represents a statistical significance at *p* < 0.05. The respective stimulated and unstimulated synaptosome pairs are color coded in each panel.

Next, we proceeded to evaluate cLTP-induced changes in surface-exposed AMPAR levels in cortical synaptosomes obtained from 8 months old male PS19 mice (expressing the human tau P301S mutation) relative to the age- and sex-matched WT control mice using the protocol of glycine priming and subsequent KCl depolarization. As expected, the basal levels of surface-exposed AMPARs in unstimulated synaptosomes were not found to be significantly altered in PS19 mice when compared to WT mice. However, while glycine priming-KCl stimulation resulted in a 19% increase in [^3^H]-AMPA binding in WT synaptosomes (*p* = 0.03; Figure 5A), cortical synaptosomes from PS19 mice failed to elicit a similar increase in surface expression of AMPARs (*p* = 0.14; Figure 5B).

## 4. Discussion

Synaptosomes are fast being recognized as efficient tools to characterize multiple aspects of mammalian synaptic physiology. These ex vivo biochemical preparations of pinched-off and resealed synaptic terminals are disconnected from the cell bodies. However, they retain much of the metabolic and enzymatic activities [11,14]. Synaptosomes are particularly of relevance in the perspective of human pathological cases, as they can be efficiently obtained from long-term stored post-mortem brain tissues with significant post-mortem delays. Not surprisingly, human synaptosomes have been employed for biochemical profiling of a wide range of synapse-specific phenomena [11,15].

The present study utilized synaptosomes obtained from both rodent and human cryopreserved brains to evaluate the postsynaptic responses upon the induction of cLTP by three different ways of chemical stimulation. Increases or decreases in surface expression of AMPARs at synaptic sites relate to the enhancements or repressions in synaptic strength in LTP and LTD, respectively. In fact, the increment of surface-expressed AMPAR levels is often regarded as a biochemical measure of LTP [5,6,7,9]. Taking advantage of this, we chose the quantification of surface AMPARs as a common readout for all the three different cLTP protocols (Figure 2). Noteworthy, surface expression of AMPARs in acute slices, primary neurons and synaptosomes have relied on membrane-impermeant radiolabeled AMPA with subsequent quantitation of surface expressed AMPARs by liquid scintillation spectroscopy [37] or autoradiography [103]. There are other methods such as surface biotinylation [45], fluorescent in situ hybridization [92] and immunolabelling followed by fluorescence- and size- sorting [39] to assess plasmalemmal AMPARs. In cell cultures, exogenous expression of fluorescently tagged AMPAR subunit GluR1 has also been employed to directly evaluate changes in its surface expression [96]. However, time and equipment constraints involved with these methods means that radiolabeling with [^3^H]-AMPA followed by liquid scintillation is still widely used for the assessment of surface AMPARs [105,109,110,111,112]. With this method, importantly, we observed similar levels of increases in AMPAR surface expression as reported in previous studies using different protocols of electrically or chemically induced LTP that employed liquid scintillation spectroscopy [37], autoradiography [103], surface biotinylation [45], fluorescent in situ hybridization using anti-AMPAR subunit antibodies [113] and live cell image of cells expressing fluorescently tagged AMPAR subunits [66].

There are many novel aspects of our study. While cLTP induction by rolipram-forskolin and TEA have been routinely performed in acute and organotypic slices and primary neurons, for the first time, this study extends the two chemical stimulations to ex vivo synaptosomes obtained from both rodent (Figure 2 A-C) and human (Figure 3) brain tissues. It should be pointed out that while we did observe statistically significant increases of synaptosomal surface expression of AMPARs using all three chemical stimulation protocols, the effects of TEA stimulation were slightly varied relative to the other two methods, particularly for human brain tissues. Nevertheless, as an NMDAR-independent LTP induction protocol (relative to the NMDAR-dependent rolipram/forskolin and glycine-KCl protocols), the TEA protocol might serve as a useful addition to the tools employed to characterize postsynaptic responses in ex vivo synaptosomes.

Another major conclusion from our study is the further evidence for the critical role of dynamic actin polymerization in mediating postsynaptic changes upon the induction of cLTP (Figure 2D-F). As the major component of synaptic cytoskeleton, actin is a key player in regulating the size and structure of the synapses. Moreover, dynamic alterations in actin polymerization status are critical for plasticity-induced remodeling of morpho-functional attributes of the synapses [58,59,63,64]. For example, actin polymerization and depolymerization are associated with spine enlargement and shrinkage during LTP and LTD, respectively [60,99,114,115]. During LTP, calcium influx through NMDARs induces a chain of molecular events resulting in fast actin polymerization, that is, the conversion of G-actin to F-actin. Activity-dependent plastic modulation of AMPAR expression on synaptic surfaces relies on such fast changes in cytoskeletal actin polymerization status [66,68,99,114,116]. Of note, ex vivo depolarization of synaptosomes has been shown to results in rapid actin polymerization [117,118]. Not surprisingly, pharmacological block of actin polymerization abolishes LTP-induced synaptic changes and AMPAR dynamics [66,68,99,101,102]. Consistent with these data, our results showing the abolishment of cLTP-induced surface expression of AMPARs by latrunculin A (Figure 2D-F) demonstrates the robustness of the synaptosomal cLTP methodologies.

Finally, we obtained the proof-of-concept of the utility of synaptosomes in the determination of cLTP changes in neurodegenerative disorders using post-mortem brain tissues from both the EOAD and FTLD cases and the PS19 mice bearing human tau P301S mutation. It should be noted here that cytoskeletal dysfunction of actin has long been associated with neurodegenerative disorders such as AD [76,77,119] and FLTD models [78,79,80]. Interestingly, widespread alterations in actin polymerization status have also been observed recently in synaptosomes obtained from AD brains [28,29]. In addition, LTP deficits are a common feature of FLTD as observed by deficiency of electrically-induced LTP in ex vivo brain slices from PS19 mice [81,120,121]. It is worth noting that only a few studies have evaluated cLTP responses in ex vivo synaptosomes obtained from animal models of AD [41,122]. Interestingly, Prieto et al. (2017) reported abnormal cLTP (using the glycine priming and KCl depolarization protocol) in synaptosomes from AD brains [41]. The present study, for the first time, demonstrated that ex vivo synaptosomes from the EOAD and FTLD cases and PS19 mice elicit deficits in cLTP responses even when the sample size was very small.

## 5. Conclusions

In conclusion, by validating two novel chemical stimulation protocols (rolipram-forskolin and TEA) in synaptosomes from rodent and human synaptosomes, we build upon the evidence for the utility of synaptosomes as an efficient tool in the apprehension of synaptic physiology and the associated molecular and cellular mechanisms associated with them, particularly in the study of clinical cases. Moreover, the elucidation of cLTP defects in both human subjects with neurodegenerative conditions of EOAD and FLTD and an animal model of FLTD suggests that this approach can be applied efficiently to study synaptic plasticity in neuropathologies. It should be noted that the clinically diagnosed FTLD cases were a mixture of FTLD-CBD (with tau inclusions) and FTLD-TDP43 (with TDP-43 inclusions). The present study is a preliminary work as part of the proof-of-concept for evaluating cLTP in synaptosomes from human and rodent brains. Additional studies with a larger sample size and multiple brain regions (for human tissue work) and age points (for animal work) are warranted to further evaluate the details of synaptic dysfunction in neurodegenerative diseases and are currently ongoing in our laboratory.

## Figures and Tables

**Table 1:** Details of the human cases used for the study.

Case No.	Clinical Diagnosis	Neuropathological Diagnosis	Gender	Age (y)	PMD (h)	Cause of Death
810		NOR	F	81	23.5	Cardiorespiratory arrest
1431		NOR	F	95	20	Respiratory failure, stroke, cardiac insufficiency and diabetes mellitus
1468		NOR	M	64	10	Exsanguination
1541		NOR	F	56	14.4	Respiratory failure
1557		NOR	M	86	7.4	Respiratory insufficiency, pulmonary metastasis
Mean ± SEM				76.4 ± 7.17	15.1 ± 3.00	
433	EOAD	AD	M	67	7.5	Cardiorespiratory arrest
685	EOAD	AD	F	68	6.5	Bronchoaspiration and senile dementia
687	EOAD	AD	M	68	5.0	Cardiorespiratory arrest and Alzheimer’s disease
709	EOAD	AD	F	63	9.0	Cardiorespiratory arrest and bronchoaspiration
806	EOAD	AD	M	67	6.0	Acute respiratory failure
Mean ± SEM				76.4 ± 7.17 ^#^	6.80 ± 0.68 ^#^	
377	FTLD	FTLD-TDP43 (Type C)	M	73	5.0	Respiratory insufficiency
697	FTLD	FTLD-CBD	M	67	10.2	Multi-organ failure
820	FTLD	FTLD-TDP43 (Type B)	F	74	5.3	Asystole
866	FTLD	FTLD-TDP43 (Type A)	F	88	6.5	Cardiorespiratory arrest
906	FTLD	FTLD-CBD	F	63	7.4	Cardiorespiratory arrest
Mean ± SEM				73.0 ± 4.25 ^#^	6.88 ± 0.94 ^#^	

^#^ No statistical differences with the NOR group (Kruskal-Wallis test with post-hoc intergroup comparisons with Dunn’s test).

## Data Availability

The data presented in this study are available in the article.
